# Complete response of advanced esophageal squamous cell carcinoma with first-line chemotherapy combined with immunotherapy: a Case report

**DOI:** 10.3389/fimmu.2025.1511663

**Published:** 2025-03-05

**Authors:** Yahong Wei, Shaohui Han, Yabin Shi, Yaxing Li, Qi Zhang, Lijuan Zhang, Yan Cheng, Xiaolu Yan, Yitao Jia

**Affiliations:** ^1^ Department of Chemotherapy Center, Hebei General Hospital, Shijiazhuang, Hebei, China; ^2^ Department of Thoracic Surgery, Hebei General Hospital, Shijiazhuang, Hebei, China; ^3^ Department of Pathology, Hebei General Hospital, Shijiazhuang, Hebei, China; ^4^ Department of Oncology, Hebei General Hospital, Shijiazhuang, Hebei, China

**Keywords:** esophageal squamous cell carcinoma (ESCC), advanced cancer, complete response, chemotherapy, immunotherapy

## Abstract

Esophageal squamous cell carcinoma (ESCC) is a prevalent and aggressive malignancy, often diagnosed at an advanced stage with poor prognosis. This case report highlights the successful treatment of a patient with advanced ESCC, who presented with bilateral lung and adrenal gland metastases. Despite the aggressive nature of the disease, the patient underwent a regimen of first-line chemotherapy combined with immunotherapy, followed by immune maintenance therapy. Remarkably, the patient achieved complete remission after the completion of treatment, demonstrating the potential efficacy of this combined therapeutic approach in managing advanced ESCC with multiple metastatic sites.

## Introduction

Esophageal cancer is recognized globally as a highly lethal malignancy, occupying the seventh position in incidence and sixth in mortality, as outlined in the latest GLOBOCAN data (1). In China, it is characterized by a high incidence of esophageal squamous cell carcinoma (ESCC), with 224,000 new cases and 187,500 deaths reported in 2022 (2). The asymptomatic presentation of early-stage ESCC frequently results in delayed diagnosis and, consequently, a poor prognosis for patients. Traditional chemotherapy for advanced ESCC has limited efficacy and high toxicity, with a median overall survival of 8-11 months (3–5). However, the advent of immunotherapy has brought forth a novel therapeutic approach. Herein, we report a unique case of a stage IV ESCC patient who achieved a complete response following a regimen of chemotherapy combined with immunotherapy, highlighting a rare and promising outcome in the treatment of this aggressive disease.

## Case description

### Patient information

A 71-year-old male patient presented to our hospital with a one-month history of anorexia and a three-day history of esophageal cancer detected during a routine physical examination. Medical History: The patient has a history of hypertension for more than five years and currently has stable blood pressure control with oral antihypertensive medication. Our patient has no history of diabetes or coronary heart disease, and no psychosocial abnormalities were observed. Personal History: The patient has a smoking history of over 40 years, consuming approximately 20 cigarettes per day. There is no history of alcohol abuse, nor any exposure to radioactive substances or poisons. Family History: There is no record of related diseases and no history of hereditary tumors in the patient’s family. Physical examination: The patient maintains good oral health, no oral mucositis or ulcers, and superficial lymph nodes were not palpably enlarged. Cardiopulmonary and abdominal examinations did not reveal any significant abnormalities. Socioeconomic Status: The patient is a retired employee of a power supply company and enjoys medical insurance, indicating a stable socioeconomic status.

### Clinical findings

The patient reported experiencing anorexia with no discernible precipitating factors since February 2023. A thoracic computed tomography (CT) examination, conducted on February 19, 2023, unveiled a suspicious esophageal lesion in the inferior segment, which was absent in a prior CT scan acquired in June 2022. Furthermore, the imaging study identified novel solid nodules in the lateral basal segment of the left inferior lung lobe, alongside enlarged solid nodules in both the lateral basal segment and the posterior segment of the right inferior lung lobe. Diffuse solid micronodules were also noted within both lungs, hinting at a high probability of intrapulmonary metastatic neoplasia. Lastly, the presence of bilateral adrenal gland metastases could not be definitively excluded.

### Diagnostic procedures

**Figure 1:**
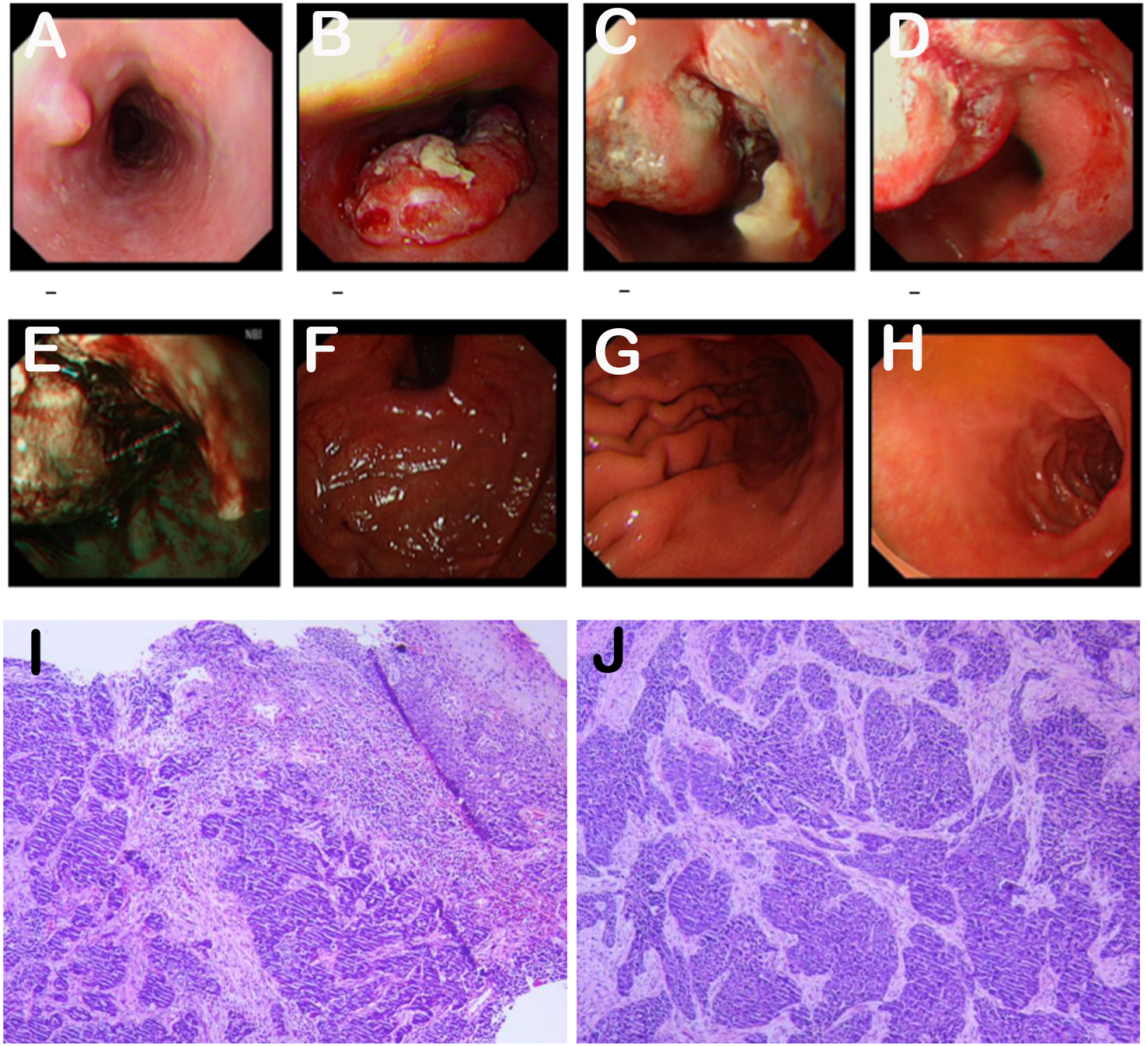
**(A-H)**. The EGD examination revealed an ulcerated lesion, involving two-thirds of the esophageal circumference. **(I, J)**. The pathological examination indicated an infiltrative poorly differentiated carcinoma, with a consideration of squamous cell carcinoma. Hematoxylin and eosin (H&E) staining. Original magnification: **(I)** ×40. **(J)** ×100.

On March 2, 2023, an esophagogastroduodenoscopy (EGD) revealed an ulcerated lesion, spanning 36-43 cm, involved two-thirds of the esophageal circumference with a narrow lumen, foul-smelling coating, and easy bleeding. NBI showed abnormal microvascular patterns, leading to the collection of five fragile biopsy samples. Tumor invasion was noted at the cardia with an unclear dentate line (**Figures 1A–H**). Endoscopic diagnosis: esophageal cancer. Pathological examination confirmed infiltrative, poorly differentiated carcinoma (consider squamous cell carcinoma)(**Figures 1I–J**), with HER2(1+) on immunohistochemical staining.

Due to the high cost of genetic testing and its exclusion from medical insurance coverage, the patient did not undergo relevant genetic testing.

### Imaging and laboratory findings

**Figure 2:**
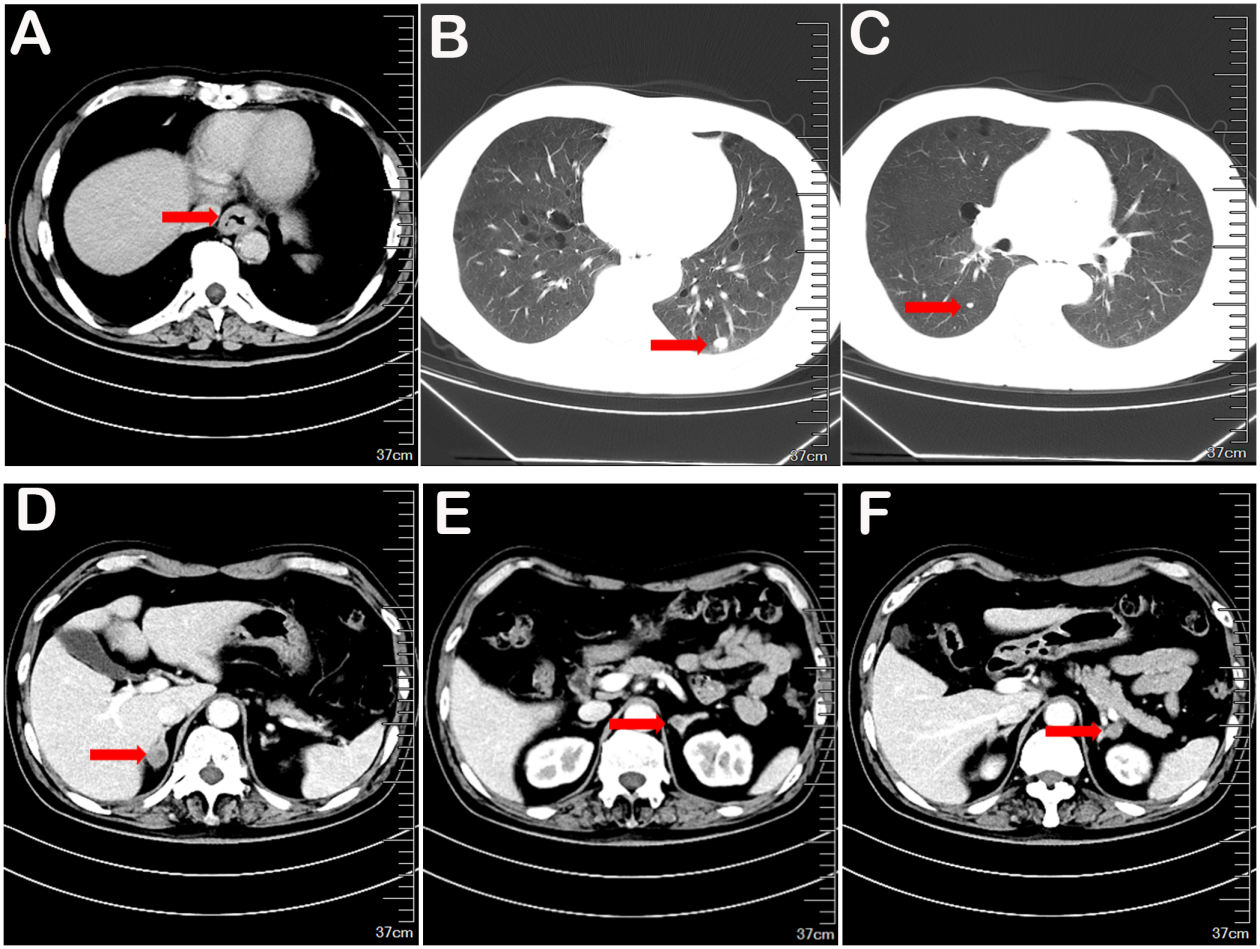
The contrast-enhanced CT scan of the chest revealed thickening of the lower esophageal segment, accompanied by new solid nodules in the outer basal segment of the left lower lung lobe and the dorsal segment of the right lower lung lobe, as well as the possibility of bilateral adrenal metastasis **(A-F)**.

On March 6, 2023, enhanced CT scans of the head, chest, and abdomen revealed no cranial metastases but confirmed the presence of lower esophageal cancer, possibly involving the cardia wall. Solid nodules were observed in the lower lobes of both lungs, accompanied by scattered micronodules throughout. Bilateral adrenal gland metastases were suspected (**Figure 2**). Bone scans were negative for metastatic involvement. Tumor markers CEA, CA199, and SCC were all within normal limits. The primary diagnosis was Stage IV (cTxN0M1) lower thoracic esophageal squamous cell carcinoma, with metastases to both lungs and the adrenal glands.

### Therapeutic intervention

**Figure 3:**
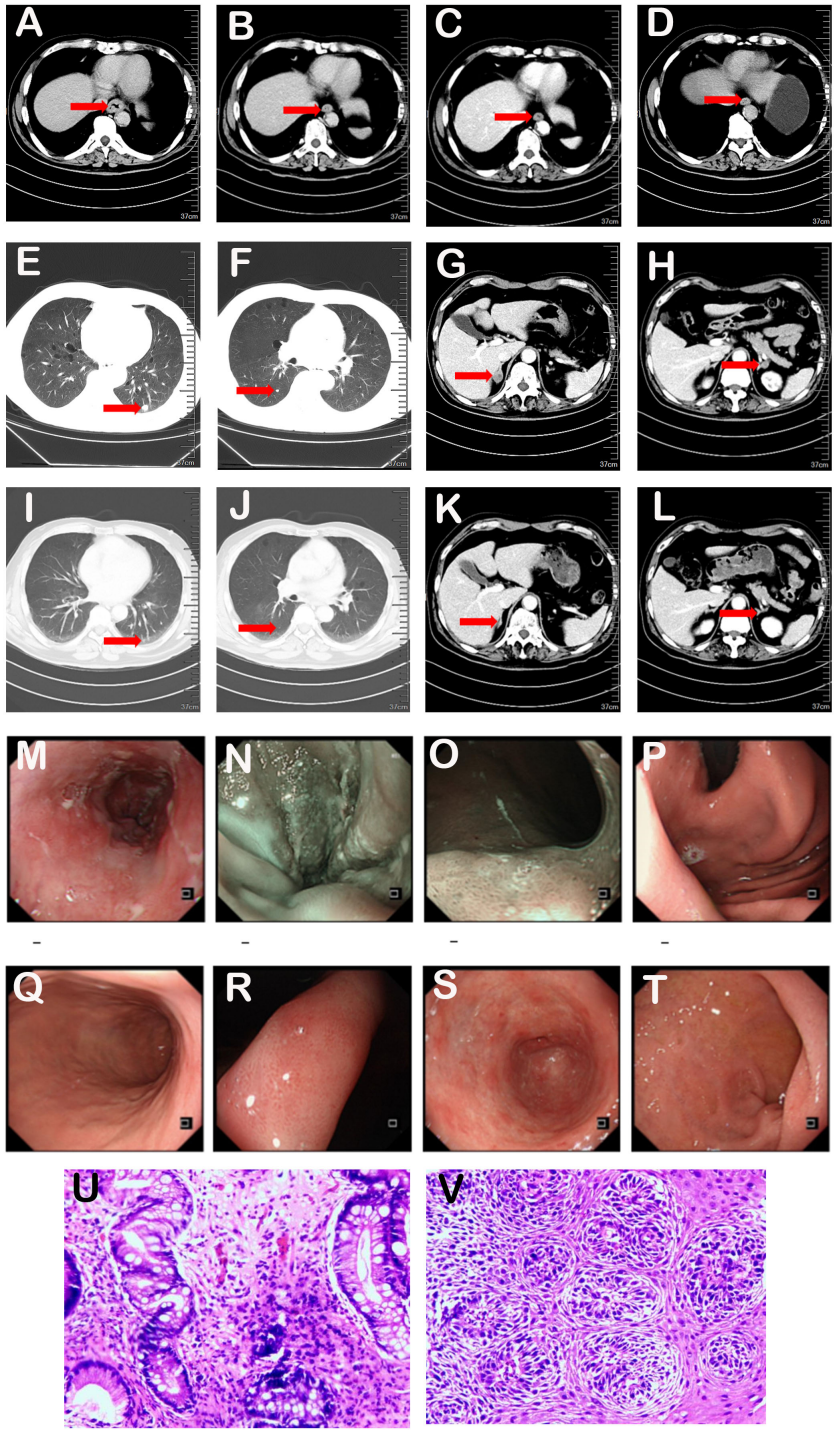
**(A-L)**. Computed tomography images before and after treatment. Review after chemotherapy combined with immunotherapy showed significant tumor reduction in lower esophagus and metastasis disappearance in lungs and adrenal glands. **(A-D)** showed lesions in the lower esophagus. The time of CT was **(A)** 2023-03-06, **(B)** 2023-5-10, **(C)** 2023-09-04, **(D)** 2024-8-28. **(E-L)** displayed metastatic lesions in both lungs and bilateral adrenal gland lesions. The time of CT was **(E-H)** 2023-03-06, **(I-L)** 2023-5-10. **(M-T)**. Gastroscopy showed rough mucosa with white plaque in lower esophagus. Patchy erythema appeared brownish-tan under NBI. **(U, V)**. Pathological examination indicated moderate chronic inflammation with intestinal metaplasia in the cardia and high-grade intraepithelial neoplasia in residual squamous cells at the biopsy sites.

From March 8, 2023, to June 28, 2023, the patient underwent six cycles of combination therapy comprising pembrolizumab (200 mg IV on Day 1), fluorouracil (1 g IV from Days 1-5), and cisplatin (40 mg IV from Days 1-3). After three cycles, a partial response (PR) was noted, characterized by a substantial reduction in the primary esophageal tumor and the disappearance of nodules in the lower lobes of both lungs and bilateral adrenal gland metastases. This PR status persisted during the maintenance therapy of immunotherapy (**Figures 3A–L**). The patient exhibited good tolerability to the treatment, without notable bone marrow suppression or impairment of liver and kidney function, and no oral mucositis, ulcers, or other complications were observed during treatment.

### Follow-up and outcomes

From July 19, 2023, to September 19, 2024, the patient underwent maintenance immunotherapy with pembrolizumab (200 mg) for a total of 20 cycles (**Table 1**). Throughout this period, the patient intermittently experienced pruritus, which was managed effectively with ebastine (10 mg orally once nightly) and a lotion containing halometasone.

**Table 1:** The duration of medication administration.

Time	Drug
2023-03-03	Pembrolizumab 200mg d1, Cisplatin 40mg d1-3, Fluorouracil 1g d1-5
2023-03-29	Pembrolizumab 200mg d1, Cisplatin 40mg d1-3, Fluorouracil 1g d1-5
2023-04-19	Pembrolizumab 200mg d1, Cisplatin 40mg d1-3, Fluorouracil 1g d1-5
2023-05-12	Pembrolizumab 200mg d1, Cisplatin 40mg d1-3, Fluorouracil 1g d1-5
2023-06-02	Pembrolizumab 200mg d1, Cisplatin 40mg d1-3, Fluorouracil 1g d1-5
2023-06-28	Pembrolizumab 200mg d1, Cisplatin 40mg d1-3, Fluorouracil 1g d1-5
2023-07-19	Pembrolizumab 200mg
2023-08-12	Pembrolizumab 200mg
2023-09-06	Pembrolizumab 200mg
2023-09-27	Pembrolizumab 200mg
2023-10-19	Pembrolizumab 200mg
2023-11-09	Pembrolizumab 200mg
2023-11-30	Pembrolizumab 200mg
2023-12-21	Pembrolizumab 200mg
2024-01-16	Pembrolizumab 200mg
2024-02-06	Pembrolizumab 200mg
2024-02-28	Pembrolizumab 200mg
2024-03-22	Pembrolizumab 200mg
2024-04-15	Pembrolizumab 200mg
2024-05-09	Pembrolizumab 200mg
2024-06-04	Pembrolizumab 200mg
2024-06-22	Pembrolizumab 200mg
2024-07-17	Pembrolizumab 200mg
2024-08-08	Pembrolizumab 200mg
2024-08-29	Pembrolizumab 200mg
2024-09-19	Pembrolizumab 200mg

### Most recent evaluation

A follow-up esophagogastroduodenoscopy (EGD) performed on September 20, 2024, revealed a rough mucosa with partial white coating in the lower esophagus (**Figures 3M–T**). Biopsies were collected from suspicious areas. Pathological analysis indicated moderate chronic inflammation with intestinal metaplasia in the cardia and high-grade intraepithelial neoplasia in residual squamous cells at the biopsy sites (**Figures 3U, V**). Notably, the primary tumor achieved a pathological complete response (pCR), while metastatic sites exhibited a clinical complete response (cCR).

### Long-term follow-up plan

We have outlined a detailed long-term follow-up plan for the patient to ensure continuous monitoring for potential relapse or late-onset side effects. This includes regular blood tests to assess blood counts, liver and kidney function, thyroid function, cortisol levels, and myocardial enzymes, allowing for early detection of immunotherapy-related adverse events. Additionally, periodic chest and abdominal CT scans, endoscopies, molecular tumor marker and esophageal radiographs will be conducted to promptly identify any signs of disease progression. Based on these assessments, adjustments to the treatment plan will be made to maximize patient benefit.

## Discussion

Esophageal cancer ranks as one of the most aggressive malignancies within the digestive system. Globally, it holds the 10th position in terms of cancer incidence and the 6th position in mortality (1). China faces a particularly heavy burden of esophageal cancer, with the annual number of new cases and deaths accounting for more than half of the global totals (6).

For advanced esophageal cancer, a multidisciplinary approach with a focus on systemic therapy is emphasized. Prior to the advent of immunotherapy, the standard first-line treatment regimen for advanced esophageal cancer was represented by two-drug combinations such as fluorouracil with cisplatin or paclitaxel with platinum-based agents. Upon disease progression, options for second-line therapy were limited (e.g., irinotecan, docetaxel), often with response rates below 10% (7, 8). Even in first-line settings, median overall survival (OS) with platinum-based standard chemotherapy was less than one year (9, 10). Besides standard chemotherapy, drugs recommended in guidelines for advanced esophageal cancer also include trastuzumab, ramucirumab, anlotinib, and apatinib. Among these, trastuzumab is recommended for first-line use in combination with FP (fluorouracil and cisplatin) in HER2-positive esophageal or esophagogastric junction (EGJ) adenocarcinoma, while the others are recommended for second-line or higher treatment due to limited patient benefit. Overall, advanced esophageal cancer faces poor treatment outcomes and limited therapeutic options, posing a significant clinical challenge.

Recently, immune checkpoint inhibitors (ICIs) have brought new opportunities for the treatment of esophageal cancer. Initially, in the later-line setting, based on studies such as KEYNOTE-181 (Phase III) (7), ESCORT (Phase III) (8), and ATTRACTION-3 (Phase III) (11), PD-1 inhibitors monotherapy has been included in guidelines as a Category 1 recommendation (1A) for second-line treatment of advanced esophageal cancer. Subsequently, with the publication of pivotal studies such as KEYNOTE-590 (12) and CHECKMATE-648 (13), the NCCN and CSCO esophageal cancer guidelines were promptly updated, with chemotherapy combined with immunotherapy becoming the standard first-line treatment for advanced esophageal cancer. In the global, multicenter, Phase III randomized trial KEYNOTE-590, which enrolled 749 patients with esophageal and EGJ cancer, more than 70% had squamous cell carcinoma, and over 90% had metastatic esophageal cancer. Pembrolizumab in combination with chemotherapy significantly improved OS compared to chemotherapy alone. Another study related to this one is the Checkmate 648 study. The CheckMate 648 study was a phase 3 trial randomizing 970 untreated adults with advanced esophageal squamous cell carcinoma to nivolumab plus chemotherapy, nivolumab plus ipilimumab, or chemotherapy alone. After 13 months’ follow-up, nivolumab combinations significantly improved overall survival (OS) and progression-free survival (PFS) vs. chemotherapy, especially in patients with PD-L1 expression ≥1%. In the overall population, median OS was also longer with nivolumab combinations. No new safety signals were identified.

The case presented herein further exemplifies the potential of ICIs in the treatment of late-stage ESCC. Our patient, initially diagnosed with stage IV ESCC, achieved a remarkable outcome after undergoing pembrolizumab-based immunotherapy in combination with chemotherapy, followed by pembrolizumab maintenance therapy. Specifically, the patient experienced a complete pathological response at the primary tumor site and a clinical complete response in metastatic lesions. This outcome is particularly noteworthy given the historically poor response rates observed in advanced ESCC.

The success of our patient’s treatment aligns with the findings of the KEYNOTE-590 study, which showed that the pembrolizumab plus chemotherapy regimen significantly prolonged OS and progression-free survival (PFS) across various patient subgroups, including those with high PD-L1 combined positive scores (CPS). Our patient’s durable response and ongoing survival beyond the median follow-up period in the KEYNOTE-590 study suggest that ICIs, particularly in combination with chemotherapy, may offer a meaningful survival benefit and improved quality of life for patients with advanced ESCC.

The complete response in this ESCC case may involve complex interactions between chemotherapy and immunotherapy (14). Chemotherapy triggers immunogenic cell death (ICD), reducing the tumor burden and enhancing immune recognition of tumor antigens. Some chemotherapy drugs also modulate the immune system, promoting immune cell activation and proliferation. Chemotherapy improves the tumor microenvironment by decreasing immunosuppressive cells, indirectly enhancing immunotherapy. Immunotherapy boosts the anti-tumor immune response, aiding chemotherapy’s effectiveness. Together, these mechanisms form a complex network for effective ESCC treatment.

Moreover, the case highlights the importance of individualizing treatment plans based on patient characteristics and disease burden. While the KEYNOTE-590 study provides a robust evidence base for the use of pembrolizumab in combination with chemotherapy, clinical decision-making should also consider patient tolerance, comorbidity profiles, and prior treatment histories. In our patient, the decision to initiate and maintain pembrolizumab therapy was guided by careful monitoring of immune-related adverse events and ongoing assessment of tumor response.

This case report has its limitations. While the treatment outcome is encouraging, it stems from a single case, and tumor heterogeneity and individual differences among patients may prevent generalization of these findings. Therefore, further research is essential to validate these observations and establish a more comprehensive understanding of the efficacy and safety of this combined therapeutic approach in ESCC.

## Conclusion

This case study exemplifies the favorable efficacy of pabolizumab-based immunotherapy, in conjunction with chemotherapy, in the management of advanced esophageal squamous cell carcinoma (ESCC). While challenges remain in achieving durable responses and managing treatment-related toxicities, the ongoing development of novel immunotherapies and their integration into multidisciplinary treatment approaches hold promise for improving outcomes for patients with this aggressive disease. Future research should focus on identifying predictive biomarkers for ICI response and exploring combination strategies to further enhance therapeutic efficacy.

## Data Availability

The original contributions presented in the study are included in the article/supplementary material. Further inquiries can be directed to the corresponding author.
